# Crystal structure of the coordination compound of triiodidomethyltin(IV) with 2,2′-bi­pyridine, MeSnI_3_·bipy

**DOI:** 10.1107/S2056989015022975

**Published:** 2016-01-01

**Authors:** Hans Reuter, Martin Reichelt

**Affiliations:** a Institute of Chemistry of New Materials, University of Osnabrueck, Barbarstrasse 7, 49069 Osnabrueck, Germany

**Keywords:** crystal structure, methyl­tin(IV) triiode, 2,2′-bi­pyridine, coordination compound

## Abstract

The title compound, (2,2′-bi­pyridine-κ^2^ *N*,*N*′)tri­iodido­meth­yltin(IV), [Sn(CH_3_)I_3_(C_10_H_8_N_2_)], crystallizing in the non-centrosymmetric ortho­rhom­bic space group *Pca*2_1_ as an inversion twin, represents one of the few structurally characterized coordination compounds of an organotin(IV) trihalide with 2,2′-biypridine. Its distorted octa­hedral geometry shows a meridional arrangement of the I atoms and the methyl group is in-plane with the five-membered chelate ring. Asymmetric bonding of the biypridine ligand to the tin(IV) atom is reflected by different Sn—N bond lengths [2.268 (4) Å *versus* 2.293 (4) Å] and caused by the static *trans* effect of the methyl group. Sn—I bond lengths show some differences with respect to their orientation to the methyl group or the bi­pyridine ligand, respectively. Angular distortions in the coordination sphere of the Sn^IV^ atom mainly arise from the large I atoms. Distortion of the 2,2′-bi­pyridine ligand as a result of its coordination to the Sn^IV^ atom are described by the twisting angle of 2.5 (2)° between the least-squares planes of the two pyridine rings, as well as by the angle of 6.2 (2)° between the two lines through the pyridine-connecting C atoms and the *para*-orientated C atoms. Directional inter­molecular inter­actions are restricted to weak I⋯H van der Waals contacts.

## Abstract 2

The title complex is one of the few structurally characterized coordination compounds of an organotin(IV) trihalide with 2,2′-biypridine. Its distorted octa­hedral geometry shows a meridional arrangement of the I atoms and the methyl group is in-plane with the five-membered chelate ring. Directional inter­molecular inter­actions are restricted to weak I⋯H van der Waals contacts.

## Chemical context   

Tin(IV) halides and organotin(IV) halides, *R*_4-*n*_SnHal_*n*_ with *n* = 1,2,3,4 and Hal = F, Cl, Br, I, show a graduated Lewis acid activity towards Lewis bases. As early as 1898, Werner and Pfeiffer stated that the acidity is decreased in the sequence: SnHal_4_ > *R*SnHal_3_ > *R*_2_SnHal_2_ > *R*_3_SnHal (Werner & Pfeiffer, 1898 ▸). Although monoorganotin(IV) halides show the highest Lewis acidity among the organotin(IV) halides, only a few complexes have been prepared and even fewer have been structurally characterized, in contrast to the situation in case of diorganotin(IV) dihalides. The few examples that have been structurally investigated are dominated by monodentate Lewis bases with O or N as coordination donors, whereas corresponding bidentate ligands are inadequately represented. Currently, there are only five coordination compounds of monoorganotin(IV) trihalides with bidentate *N*,*N*-chelating ligands listed in the Cambridge Crystallographic Database (Version 5.36, last update May 2015; Groom & Allen, 2014 ▸) but only three, BzlSnCl_3_(phen) (Hall & Tiekink, 1996 ▸), **1**, EtSnI_3_(bipy) (Paseshnichenko *et al.*, 1984 ▸), **2**, *R*′SnCl_3_(bipy) with *R*′ = 3-(4-meth­oxy­benz­yl)cyclo­penta­dienyl (Gleeson *et al.*, 2008 ▸), **3**, exhibit an almost planar backbone of the ligand as is characteristic for 2,2′-bi­pyridine (bipy) or 1,10-phenanthroline (phen). From a fundamental point of view, such complexes are of special inter­est, because of two possible steroisomers which differ in the position of the organic substituent in relation to the plane of the ligand (in-plane or perpendicular) while the three halide atoms adopt a meridional or facial orientation. The majority of all complexes investigated exhibit a meridional arrangement of the halide atoms, only **3** features a facial one.
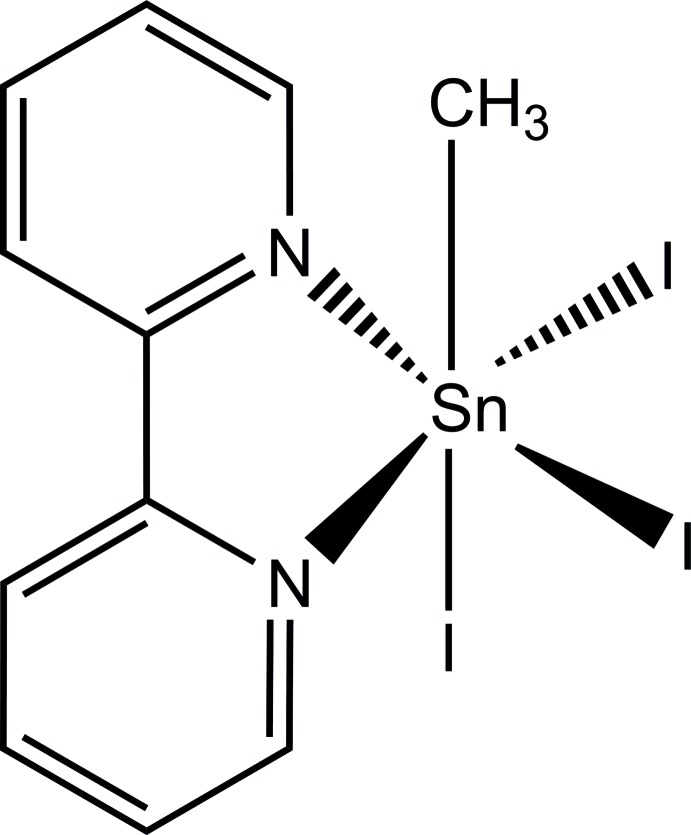


During a systematic study (Reuter *et al.*, 2011 ▸) on the solid-state structures of diorganotin(IV) dihalides, *R*_2_SnHal_2_, we were inter­ested in methylphenyldiiodidotin(IV), MePhSnI_2_, because of the unique crystal structure of the corresponding dichloride (Amini *et al.*, 1987 ▸). Experiments to achieve this diiodide from the corresponding oxide by reaction with aqueous ammonium iodide, however, failed as the resulting liquid turned out to be a mixture of two or more different unknown organotin species which could not purified by distillation. We therefore tried to synthesize derivates of these compounds by adding 2,2′-bi­pyridine to the mixtures in the hope of obtaining single crystals for identification. Indeed, the synthesis succeeded and we found two different kinds of single crystals, orange needles of the title compound and red blocks of the 2,2′-bi­pyridine complex of dimethyldiiodidotin(IV), Me_2_SnI_2_·bipy, the structure of which was also confirmed by X-ray diffraction (Reuter & Reichelt, unpublished results).

## Structural commentary   

**Figure 1:**
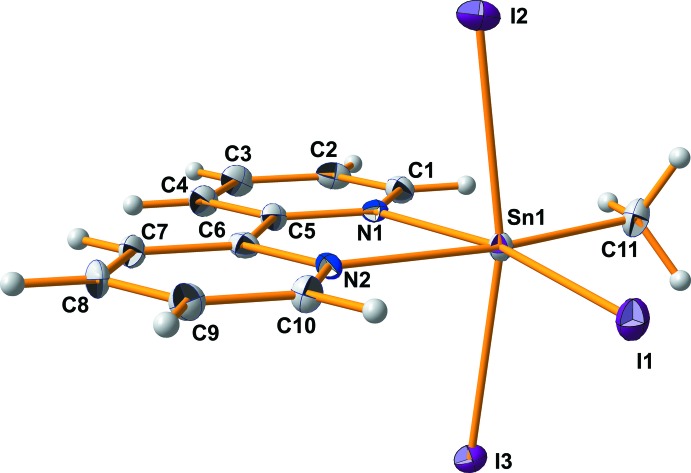
Thermal ellipsoid model of the asymmetric unit in the crystal structure of the title compound with the atomic numbering scheme used. With exception of the H atoms, which are shown as spheres of arbitrary radius, all other atoms are drawn with displacement ellipsoids at the 50% probability level.

The tin(IV) atom of the title compound is distorted octa­hedrally coordinated with the methyl group in plane with the chelating ligand and the iodine atoms in a meridional arrangement (Fig. 1 ▸). Although the formation of the five-membered chelate ring between the bidentate 2,2′-biypridine ligand and the tin(IV) atom provides the complex a certain rigidity, there remains enough conformational adaptability to react flexibly towards electronic as well as steric intra­molecular or inter­molecular demands. Since the pioneering work of Buslaev *et al.* (1989 ▸), the important role of electronic effects on bond lengths in complexes of monoorganontin(IV) halides in particular and monoorganotin(IV) compounds in general (Reuter & Ye, 2013 ▸; Reichelt & Reuter, 2013 ▸) is well established and introduced into the literature as the static *trans-effect* meaning that a bond *trans* to the organic group is shortened in comparison to a comparable bond in *cis* position. As a result, the 2,2′-bi­pyridine ligand of the title compound bonds asymmetrically to the tin(IV) atom: the Sn—N bond *trans* to the methyl group [*d*(Sn—N) = 2.268 (4) Å] is shorter than the other [*d*(Sn—N) = 2.293 (4) Å].

The lengths of the three Sn—I bonds are very similar, although there is a significant difference between the Sn—I bond *trans* to the bi­pyridine ligand [*d*(Sn1—I1) = 2.8041 (5) Å] and the two *cis* orientated Sn—I bonds [mean value: *d*(Sn—I) = 2.853 (7) Å]. Similar Sn—I bond lengths (2.808, 2.838–2.878 Å) are found in the ethyl compound **2**. All in all, these Sn—I bonds of sixfold-coordinated tin(IV) are about 0.2 Å longer than for the tin(IV) atom in the tetra­hedral environment of SnI_4_, where a mean value of 2.661 Å has been observed (Reuter & Pawlak, 2001 ▸).

In comparison with the Sn—C bond length of the corres­ponding dimethyldiiodidotin(IV) compound [2.122 (3) Å; Reuter & Reichelt, unpublished], that of the title compound is rather long [2.179 (5) Å]. In the corresponding ethyl compound **2**, the bond is even longer (2.199 Å). Whether this reflects a general trend is difficult to decide, because no other precise structural data of complexes of monoorganotin(IV) triiodides are available.

**Figure 2:**
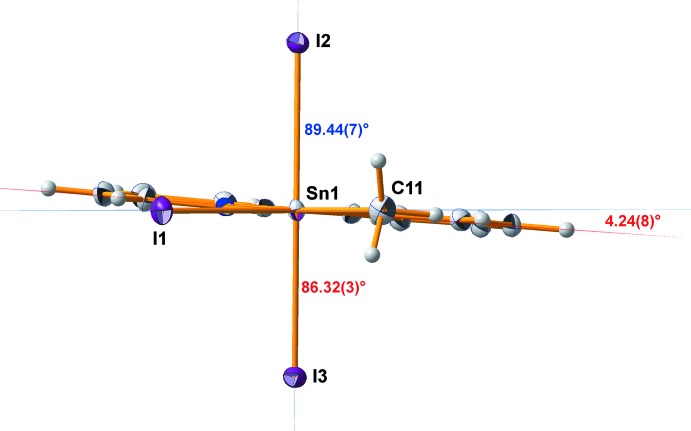
Displacement ellipsoid model of the title compound looking down the direction of the planes I1–Sn1–C11 and I2–Sn1–I3 and their dihedral angle (all in blue); orientation of the least-squares plane through all atoms of the 2,2′-bi­pyridine ligand in relation to these planes with the corresponding dihedral angles (all in red).

Other distortions of the octa­hedral coordination around the central tin(IV) atom concern bond angles which deviate significantly from the bond angles in a regular octa­hedron. The distortions are caused mainly by the large iodine atoms, which demand the most space in the environment of the tin atom, with the result that the bond angles between the iodine atoms themselves, as well as the bond angles between the iodine atoms and the methyl group, are significantly larger than 90° [96.9 (1), 94.9 (2), 93.06 (1)°; Table 1 ▸]. As a consequence, the axis through the iodine atoms *cis* to the bipy ligand is bent [167.04 (2)°] in direction of the chelate ligand. All these bond-angle distortions, however, take place within the planes these four atoms are involved in [I1–Sn1–C11/I2–Sn1–I3] so that these planes are almost perpendicular to each other [dihedral angle: 89.44 (7)°] (Fig. 2 ▸). In contrast, the bi­pyridine ligand adopts an inclined orientation [dihedral angles: 86.32 (3)° with I2–Sn1–I3; 4.24 (8)° with I1–Sn1–C11].

**Figure 3:**
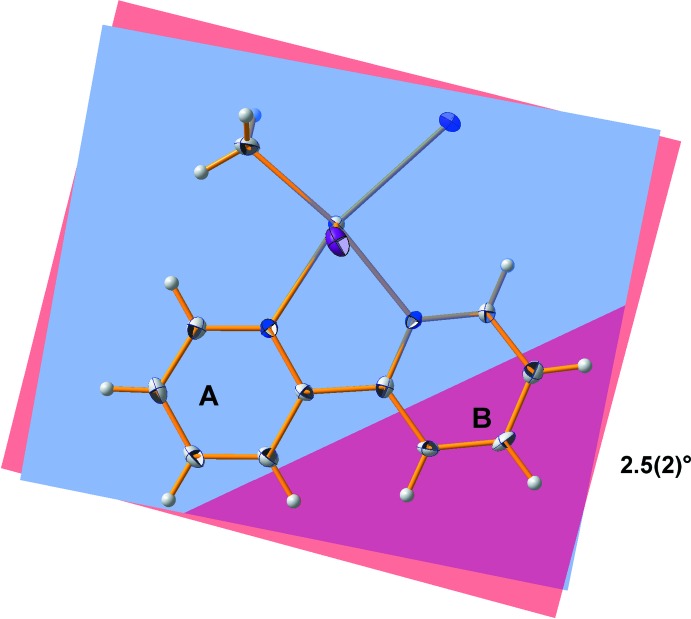
Displacement ellipsoid model of the title compound looking onto the biypridine ligand, showing its twisting described by the dihedral angle between the least-squares planes through the two pyridine moieties of this ligand.

**Figure 4:**
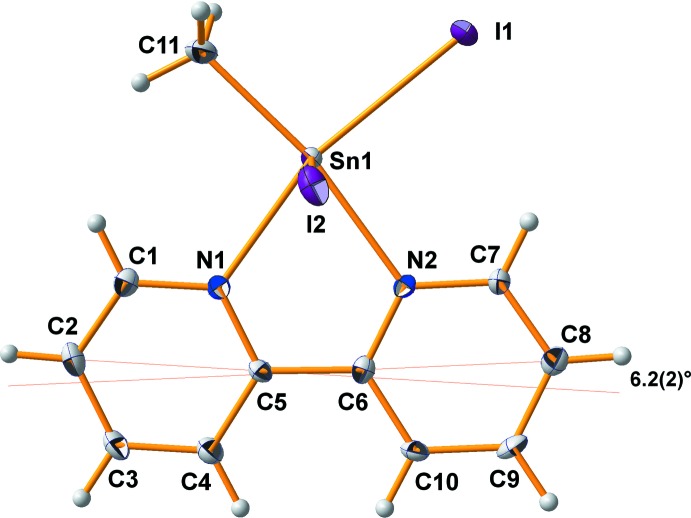
Displacement ellipsoid model of the title compound looking onto the bi­pyridine ligand, showing its bending described by the angle between the two lines through the connecting and the *para-*oriented carbon atoms.

The bi­pyridine ligand itself shows the typical bond lengths and angles: *d*(C—N) = 1.346, *d*(C—C)_arom_ = 1.385, C—N—C = 119.3°, all mean values. As usual, the shorter C—N bonds and angles at nitro­gen are compensated by larger bond angles at the carbon atoms so that planarity of both pyridine moieties is retained [deviations from least-squares plane (Å): N1 = −0.005 (3), C1 = −0.002 (3), C2 = 0.005 (4), C3 = −0.001 (4), C4 = −0.007 (3), C5 = 0.010 (3); N2 = −0.002 (3), C7 = 0.004 (3), C8 = −0.003 (3), C9 = 0.001 (3), C10 = 0.001 (3), C6 = −0.001 (3)]. The C—C single bond between the two pyridine groups has a length of 1.488 (6) Å. The inter­action of the ligand with the tin(IV) atom, however, produces some distortions affecting the planarity of the ligand as a whole, as well as the orientation of the two pyridine rings in relation to each other. Twisting of the bi­pyridine ligand is best described by the dihedral angle of 2.5 (2)° (Fig. 3 ▸) between the least-squares planes of the pyridine rings, while its bending (Fig. 4 ▸) can be qu­anti­tatively described using the angle of 6.2 (2)° between the lines through the linking carbon atoms (C5 and C6) and their *para*-orientated counterparts (C2 and C8).

## Supra­molecular features   

**Figure 5:**
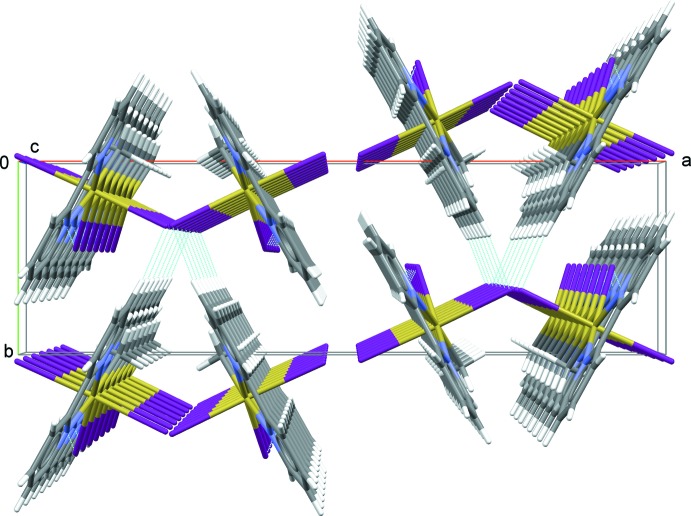
Perspective view of the crystal structure seen parallel to [001], looking down the strands resulting from short I⋯H van der Waals contacts between bi­pyridine and iodine of neighbouring complexes. All van der Waals contacts shorter 3.2 Å are drawn as dashed blue lines.

In the solid state, there are only weak inter­actions between the complexes. No π–π inter­actions between the aromatic rings or Sn⋯I inter­actions between neighboring mol­ecules are observed (Fig. 5 ▸). I⋯H van der Waals type contacts are the only type of directional inter­actions between mol­ecules. The shortest inter­action [3.047 Å] is found between an H atom (H10) of the bi­pyridine ligand of one mol­ecule with an iodine atom (I1) of the neighboring mol­ecule almost colinear with the *c* axis (Fig. 5 ▸). Because of space-group symmetry, the strands of mol­ecules connected this way are arranged in V-shaped pairs [opening angle about 52°] with an offset of *c*/2 between individual I⋯H connected strands. Along the *b-*axis direction, neighboring pairs of mol­ecules are connected *via* somewhat longer I⋯H contacts [3.162 Å, I3⋯H2], while along the *a*-axis direction there are no contacts shorter than 3.2 Å.

## Synthesis and crystallization   

In a typical experiment, a suspension of 4.1 g (18 mmol) MePhSnO and 5.6 g (50 mmol, excess) NH_4_I in toluene was heated to reflux of the solvent for 24 h using a Soxhlet extractor filled with silica gel for water adsorption. After evaporation of the organic solvent, the remaining liquid was proved by ^13^C NMR spectroscopy to be composed of at least two different organotin(IV) species. Attempts to separate these compounds by distillation failed. Re-dissolution of the residue in toluene, and addition of 2,2′-bi­pyridine followed by slow evaporation of the organic solvent, however, resulted in the formation of two different crystal forms; orange needles of the title compound and red blocks of Me_2_SnI_2_·bipy. Unfortunately, further attempts to separate larger amounts of the different species for further characterization were unsuccessful.

## Refinement details   

Crystal data, data collection and structure refinement details are summarized in Table 2 ▸. The title compound crystallizes in the non-centrosymmetric, ortho­rhom­bic space group *Pca*2_1_. As the Flack parameter deviates significantly from zero, the structure was refined as an inversion twin with a twin-factor of 0.12 (3). All hydrogen atoms could be localized in difference Fourier syntheses but were refined in geometric positions riding on the carbon atoms with C—H distances of 0.98 Å (–CH_3_) and 0.95 Å (–CH_arom_) and with *U*_iso_(H) = 1.2*U*_eq_(C). Reflection 2 0 0 was omitted because it was affected by the beam stop.

## Supplementary Material

Crystal structure: contains datablock(s) I, New_Global_Publ_Block. DOI: 10.1107/S2056989015022975/zl2654sup1.cif

Structure factors: contains datablock(s) I. DOI: 10.1107/S2056989015022975/zl2654Isup2.hkl

CCDC reference: 1439787

Additional supporting information:  crystallographic information; 3D view; checkCIF report

## Figures and Tables

**Table 1:** Selected geometric parameters (Å, °)

Sn1—C11	2.179 (5)	Sn1—I1	2.8041 (5)
Sn1—N1	2.268 (4)	Sn1—I3	2.8476 (4)
Sn1—N2	2.293 (4)	Sn1—I2	2.8580 (4)
			
C11—Sn1—I1	96.85 (14)	I1—Sn1—I2	94.416 (13)
C11—Sn1—I3	94.87 (15)	I3—Sn1—I2	167.039 (17)
I1—Sn1—I3	93.064 (13)		

**Table 2:** Experimental details

Crystal data	
Chemical formula	[Sn(CH_3_)I_3_(C_10_H_8_N_2_)]
*M*_r_	670.61
Crystal system, space group	Orthorhombic, *P* *c* *a*2_1_
Temperature (K)	100
*a*, *b*, *c* (Å)	23.5604 (4), 7.0367 (3), 9.5792 (4)
*V* (Å^3^)	1588.11 (10)
*Z*	4
Radiation type	Mo *K*α
μ (mm^−1^)	7.42
Crystal size (mm)	0.23 × 0.15 × 0.11

Data collection	
Diffractometer	Bruker APEXII CCD
Absorption correction	Multi-scan (*SADABS*; Bruker, 2009 ▸)
*T*_min_, *T*_max_	0.285, 0.501
No. of measured, independent and observed [*I* > 2σ(*I*)] reflections	48663, 4026, 3990
*R*_int_	0.039
(sin θ/λ)_max_ (Å^−1^)	0.677

Refinement	
*R*[*F* ^2^ > 2σ(*F* ^2^)], *wR*(*F* ^2^), *S*	0.016, 0.037, 1.21
No. of reflections	4026
No. of parameters	156
No. of restraints	1
H-atom treatment	H-atom parameters constrained
Δρ_max_, Δρ_min_ (e Å^−3^)	0.61, −0.81
Absolute structure	Refined as an inversion twin.
Absolute structure parameter	0.12 (3)

Computer programs: *APEX2* (Bruker, 2009 ▸), *SAINT* (Bruker, 2009 ▸), *SHELXS97* and *SHELXTL* (Sheldrick, 2008 ▸), *SHELXL2014* (Sheldrick, 2015 ▸), *DIAMOND* (Brandenburg, 2006 ▸) and *Mercury* (Macrae *et al.*, 2008 ▸).

## supplementary crystallographic information

### Crystal data

**Table:**

[Sn(CH_3_)I_3_(C_10_H_8_N_2_)]	*D*_x_ = 2.805 Mg m^−^^3^
*M**_r_* = 670.61	Mo *K*α radiation, λ = 0.71073 Å
Orthorhombic, *P**c**a*2_1_	Cell parameters from 9469 reflections
*a* = 23.5604 (4) Å	θ = 2.7–28.8°
*b* = 7.0367 (3) Å	µ = 7.42 mm^−^^1^
*c* = 9.5792 (4) Å	*T* = 100 K
*V* = 1588.11 (10) Å^3^	Block, orange
*Z* = 4	0.23 × 0.15 × 0.11 mm
*F*(000) = 1200	

### Data collection

**Table:**

Bruker APEXII CCD diffractometer	3990 reflections with *I* > 2σ(*I*)
φ and ω scans	*R*_int_ = 0.039
Absorption correction: multi-scan (*SADABS*; Bruker, 2009)	θ_max_ = 28.8°, θ_min_ = 2.7°
*T*_min_ = 0.285, *T*_max_ = 0.501	*h* = −31→31
48663 measured reflections	*k* = −9→9
4026 independent reflections	*l* = −12→11

### Refinement

**Table:**

Refinement on *F*^2^	Secondary atom site location: difference Fourier map
Least-squares matrix: full	Hydrogen site location: inferred from neighbouring sites
*R*[*F*^2^ > 2σ(*F*^2^)] = 0.016	H-atom parameters constrained
*wR*(*F*^2^) = 0.037	*w* = 1/[σ^2^(*F*_o_^2^) + (0.0111*P*)^2^ + 2.8899*P*] where *P* = (*F*_o_^2^ + 2*F*_c_^2^)/3
*S* = 1.21	(Δ/σ)_max_ < 0.001
4026 reflections	Δρ_max_ = 0.61 e Å^−^^3^
156 parameters	Δρ_min_ = −0.81 e Å^−^^3^
1 restraint	Absolute structure: Refined as an inversion twin.
Primary atom site location: structure-invariant direct methods	Absolute structure parameter: 0.12 (3)

### Special details

**Table:**

Geometry. All e.s.d.'s (except the e.s.d. in the dihedral angle between two l.s. planes) are estimated using the full covariance matrix. The cell e.s.d.'s are taken into account individually in the estimation of e.s.d.'s in distances, angles and torsion angles; correlations between e.s.d.'s in cell parameters are only used when they are defined by crystal symmetry. An approximate (isotropic) treatment of cell e.s.d.'s is used for estimating e.s.d.'s involving l.s. planes.
Refinement. Refined as a 2-component inversion twin.

### Fractional atomic coordinates and isotropic or equivalent isotropic displacement parameters (Å^2^)

**Table:**

	*x*	*y*	*z*	*U*_iso_*/*U*_eq_	
Sn1	0.36213 (2)	0.17120 (4)	0.23984 (4)	0.01142 (6)	
C11	0.3258 (2)	−0.0290 (8)	0.3886 (6)	0.0208 (11)	
H11	0.3094	−0.1370	0.3381	0.025*	
H12	0.2960	0.0344	0.4428	0.025*	
H13	0.3555	−0.0744	0.4518	0.025*	
I1	0.38951 (2)	0.45560 (5)	0.43351 (4)	0.01814 (7)	
I2	0.47258 (2)	0.00336 (5)	0.24365 (4)	0.01947 (7)	
I3	0.25648 (2)	0.34316 (4)	0.16992 (3)	0.01643 (7)	
N1	0.39831 (16)	0.3374 (6)	0.0575 (4)	0.0115 (7)	
C1	0.4260 (2)	0.5041 (7)	0.0732 (5)	0.0145 (9)	
H1	0.4291	0.5579	0.1639	0.017*	
C2	0.4501 (2)	0.5987 (8)	−0.0381 (5)	0.0174 (10)	
H2	0.4696	0.7152	−0.0241	0.021*	
C3	0.4455 (2)	0.5221 (8)	−0.1699 (6)	0.0187 (10)	
H3	0.4615	0.5851	−0.2482	0.022*	
C4	0.4168 (2)	0.3504 (7)	−0.1865 (5)	0.0154 (9)	
H4	0.4126	0.2957	−0.2765	0.019*	
C5	0.39433 (18)	0.2602 (6)	−0.0701 (6)	0.0133 (8)	
C6	0.36488 (19)	0.0737 (6)	−0.0814 (5)	0.0126 (9)	
N2	0.34677 (16)	−0.0009 (6)	0.0402 (4)	0.0123 (8)	
C7	0.3195 (2)	−0.1687 (7)	0.0394 (6)	0.0167 (10)	
H7	0.3073	−0.2213	0.1256	0.020*	
C8	0.3088 (2)	−0.2670 (7)	−0.0824 (6)	0.0185 (10)	
H8	0.2890	−0.3846	−0.0803	0.022*	
C9	0.3276 (2)	−0.1905 (8)	−0.2087 (6)	0.0194 (10)	
H9	0.3210	−0.2559	−0.2939	0.023*	
C10	0.3558 (2)	−0.0188 (7)	−0.2079 (6)	0.0172 (10)	
H10	0.3688	0.0358	−0.2927	0.021*	

### Atomic displacement parameters (Å^2^)

**Table:**

	*U*^11^	*U*^22^	*U*^33^	*U*^12^	*U*^13^	*U*^23^
Sn1	0.01426 (13)	0.01016 (13)	0.00985 (14)	−0.00100 (10)	0.00007 (12)	−0.00058 (13)
C11	0.024 (3)	0.024 (3)	0.014 (2)	−0.001 (2)	0.003 (2)	−0.003 (2)
I1	0.02387 (15)	0.01725 (13)	0.01331 (14)	−0.00236 (13)	−0.00016 (12)	−0.00381 (14)
I2	0.01713 (13)	0.01535 (13)	0.02594 (16)	0.00278 (10)	−0.00681 (14)	−0.00460 (14)
I3	0.01422 (13)	0.01633 (14)	0.01874 (15)	0.00167 (11)	0.00060 (12)	−0.00138 (13)
N1	0.0111 (16)	0.0113 (18)	0.0123 (19)	−0.0006 (14)	−0.0006 (15)	0.0025 (15)
C1	0.015 (2)	0.013 (2)	0.015 (2)	−0.0037 (17)	0.0007 (18)	−0.0007 (19)
C2	0.017 (2)	0.015 (2)	0.020 (3)	−0.0011 (17)	0.0026 (19)	0.002 (2)
C3	0.021 (2)	0.020 (3)	0.016 (2)	0.0001 (19)	0.0066 (19)	0.007 (2)
C4	0.021 (2)	0.016 (2)	0.009 (2)	0.0008 (19)	0.0006 (18)	−0.0012 (18)
C5	0.0101 (18)	0.013 (2)	0.016 (2)	−0.0005 (15)	−0.0008 (18)	0.003 (2)
C6	0.014 (2)	0.013 (2)	0.011 (2)	0.0007 (15)	−0.0024 (17)	−0.001 (2)
N2	0.0126 (18)	0.0106 (19)	0.014 (2)	0.0025 (14)	0.0006 (16)	0.0002 (16)
C7	0.017 (2)	0.015 (2)	0.018 (3)	−0.0001 (18)	−0.0003 (19)	0.002 (2)
C8	0.019 (2)	0.013 (2)	0.023 (3)	−0.0009 (18)	−0.006 (2)	−0.004 (2)
C9	0.024 (2)	0.016 (2)	0.019 (2)	0.0002 (19)	−0.006 (2)	−0.0059 (19)
C10	0.020 (2)	0.016 (2)	0.015 (2)	0.0007 (19)	−0.0017 (18)	−0.0027 (19)

### Geometric parameters (Å, º)

**Table:**

Sn1—C11	2.179 (5)	C3—C4	1.393 (7)
Sn1—N1	2.268 (4)	C3—H3	0.9500
Sn1—N2	2.293 (4)	C4—C5	1.388 (7)
Sn1—I1	2.8041 (5)	C4—H4	0.9500
Sn1—I3	2.8476 (4)	C5—C6	1.488 (6)
Sn1—I2	2.8580 (4)	C6—N2	1.347 (7)
C11—H11	0.9800	C6—C10	1.392 (7)
C11—H12	0.9800	N2—C7	1.344 (6)
C11—H13	0.9800	C7—C8	1.379 (8)
N1—C5	1.341 (7)	C7—H7	0.9500
N1—C1	1.351 (6)	C8—C9	1.396 (8)
C1—C2	1.379 (7)	C8—H8	0.9500
C1—H1	0.9500	C9—C10	1.379 (7)
C2—C3	1.377 (8)	C9—H9	0.9500
C2—H2	0.9500	C10—H10	0.9500
			
C11—Sn1—N1	169.90 (18)	C1—C2—H2	120.4
C11—Sn1—N2	98.16 (18)	C2—C3—C4	118.8 (5)
N1—Sn1—N2	71.89 (15)	C2—C3—H3	120.6
C11—Sn1—I1	96.85 (14)	C4—C3—H3	120.6
N1—Sn1—I1	93.16 (11)	C5—C4—C3	119.4 (5)
N2—Sn1—I1	164.86 (10)	C5—C4—H4	120.3
C11—Sn1—I3	94.87 (15)	C3—C4—H4	120.3
N1—Sn1—I3	85.89 (10)	N1—C5—C4	121.3 (4)
N2—Sn1—I3	83.69 (10)	N1—C5—C6	117.2 (5)
I1—Sn1—I3	93.064 (13)	C4—C5—C6	121.5 (5)
C11—Sn1—I2	94.73 (15)	N2—C6—C10	121.4 (4)
N1—Sn1—I2	83.15 (10)	N2—C6—C5	115.4 (4)
N2—Sn1—I2	86.34 (10)	C10—C6—C5	123.2 (5)
I1—Sn1—I2	94.416 (13)	C7—N2—C6	119.3 (4)
I3—Sn1—I2	167.039 (17)	C7—N2—Sn1	123.0 (3)
Sn1—C11—H11	109.5	C6—N2—Sn1	117.7 (3)
Sn1—C11—H12	109.5	N2—C7—C8	122.2 (5)
H11—C11—H12	109.5	N2—C7—H7	118.9
Sn1—C11—H13	109.5	C8—C7—H7	118.9
H11—C11—H13	109.5	C7—C8—C9	118.8 (4)
H12—C11—H13	109.5	C7—C8—H8	120.6
C5—N1—C1	119.2 (4)	C9—C8—H8	120.6
C5—N1—Sn1	117.8 (3)	C10—C9—C8	119.0 (5)
C1—N1—Sn1	122.9 (3)	C10—C9—H9	120.5
N1—C1—C2	122.1 (5)	C8—C9—H9	120.5
N1—C1—H1	118.9	C9—C10—C6	119.3 (5)
C2—C1—H1	118.9	C9—C10—H10	120.4
C3—C2—C1	119.2 (5)	C6—C10—H10	120.4
C3—C2—H2	120.4		
